# Evolution of the hemagglutinin gene of H3N8 canine influenza virus in dogs

**DOI:** 10.1007/s11262-014-1102-8

**Published:** 2014-07-24

**Authors:** Heidi L. Pecoraro, Susi Bennett, Miranda E. Spindel, Gabriele A. Landolt

**Affiliations:** 1 From the Departments of Microbiology, Immunology, and Pathology, College of Veterinary Medicine and Biomedical Science, Colorado State University, 300 West Drake Road, Fort Collins, CO 80523-1678 USA; 2 Clinical Sciences, College of Veterinary Medicine and Biomedical Science, Colorado State University, 300 West Drake Road, Fort Collins, CO 80523-1678 USA

**Keywords:** Influenza A virus, MEME analysis, Antigenic drift

## Abstract

With the widespread use of a recently developed canine influenza virus (CIV) H3N8 vaccine, continual molecular evaluation of circulating CIVs is necessary for monitoring antigenic drift. The aim of this project was to further describe the genetic evolution of CIV, as well as determine any genetic variation within potential antigenic regions that might result in antigenic drift. To this end, the hemagglutinin gene of 19 CIV isolates from dogs residing in Colorado, New York, and South Carolina humane shelters was sequenced and compared to CIV strains isolated during 2003–2012. Phylogenetic analysis suggests that CIV might be diverging into two geographically distinct lineages. Using a mixed-effects model for evolution and single likelihood ancestor counting methods, several amino acid sites were found to be undergoing selection pressure. Additionally, a total of six amino acid changes were observed in two possible antigenic sites for CIVs isolated from Colorado and New York humane shelters between 2009 and 2011. As CIV isolates might be diverging into geographically distinct lineages, further experiments are warranted to determine the extent of antigenic drift occurring within circulating CIV.

## Introduction

Although serological evidence suggests H3N8 canine influenza virus (CIV) has been circulating in U.S. dogs since as early as 1999 [1], it was not until 2004 that the virus was isolated from Florida racing greyhounds exhibiting signs of infectious respiratory disease [2]. Early phylogenetic analyses showed CIV, an influenza A virus in the family *Orthomyxoviridae*, to be closely related to contemporary equine influenza viruses (EIV) of the Florida Clade 1 sublineage [2, 3]. From the emerging CIVs, ten amino acid substitutions within the hemagglutinin (HA), neuraminidase, nucleoprotein, and polymerase proteins were identified [3]. Unlike other horse-to-dog EIV transmission events in which dogs have been only incidentally infected [4, 5], CIV represents a new threat to canine health, as the virus rapidly spreads to naïve dogs throughout the racing track circuit [2, 6], animal shelters [2, 7, 8], and boarding facilities [9]. Despite successful experimental transmission of EIV and CIV in both horses and dogs [10–13], host restriction is currently being maintained in naturally-infected animals [14]. Furthermore, CIV isolates sequenced between 2005 and 2008 share the same ten amino acid substitutions originally identified in early Florida CIV, as well as an additional nine amino acid substitutions among the influenza proteins [14] (three within the HA alone). These findings indicate that CIV continues to evolve from both its canine and equine influenza predecessors.

Given its vital role during influenza infection, it is not surprising that the HA protein of influenza A virus is the main target for antibody attachment and virus neutralization [15, 16]. Five antigenic regions (A–E) have been identified in the HA subtype 3 (H3) HA1 subunit protein of human and equine influenza viruses [17–21], and mutations within these antigenic regions have been associated with immune escape or antigenic drift [22], which can result in vaccine failure and increased host susceptibility to influenza infection. It has been suggested that antigenic drift has epidemiological impact when four or more amino acid substitutions are located within at least two of these antigenic regions [22]. Antigenic drift has been reported in human H3 influenza viruses [19, 21], with influenza strains clustering into antigenic groups over time. For example, influenza A H3N2 viruses isolated from humans between 1968 and 2003 formed 11 distinct antigenic clusters [21]. Equine influenza viruses, in contrast, clustered into only three antigenic groups over the same time period [23], while antigenic drift has yet to be detected or described in CIVs isolated from dogs [24, 25].

With the widespread use of a recently developed killed CIV vaccine [26], continual molecular evaluation of circulating CIVs is clearly necessary for monitoring antigenic drift. Furthermore, as CIV emerged only within the last decade, it is important to continually monitor its genetic diversity and whether geographically distinct viral lineages are evolving [2, 3, 14, 27]. By analyzing the HA nucleotide and protein sequences of the published set of CIVs, as well as the newly isolated CIVs from CIV-epidemic and endemic U.S. regions, we are able to further describe the evolution of CIV since it first emerged in 2004, and report amino acid variations within potential antigenic sites that could possibly result in the first clustering of antigenically distinct CIVs.

## Materials and methods

### Animals

Dogs residing in six U.S. shelters participated in this prospective study. The six shelters were located near or within metropolitan centers (Sacramento, CA; Colorado Springs, CO; Tampa Bay, FL; New York, NY; Charlestown, SC; and Austin, TX) and were part of a larger CIV surveillance study conducted from December 2009 to January 2012 [28]. Shelters were chosen in regions that were considered CIV-endemic (CO, FL, NY), where previous studies have reported CIV outbreaks [2, 6, 27, 28], and in regions where CIV status was unknown (CA, SC, TX). Nasal swabs were taken from approximately 40 dogs every month per shelter, and all dogs, irrespective of age, breed, sex, and vaccination or health status, were considered eligible to participate. Prior to initiation, all studies were reviewed and approved by Colorado State University IACUC.

### Clinical samples

Nasal swab samples were collected from dogs on-site at each humane shelter, as previously described [8, 28]. Samples were refrigerated at 4 °C and shipped monthly to Colorado State University Veterinary Teaching Hospital (VTH). Upon arrival in the laboratory, 200 μl of nasal swab viral transport medium was removed from each sample and placed in a 96-well block for later ribonucleic acid (RNA) isolation. The remaining sample, along with the samples in the 96-well block, were immediately stored at −80 °C until further processing.

### Real-time reverse transcription-polymerase chain reaction (RT-PCR) assay

Using an automated RNA isolation system (Qiagen Qiaxtractor; Hilden, Germany), viral RNA was extracted from the 200-μl viral transport medium aliquots and eluted in a final volume of 60 μl water. A one-step real-time RT-PCR assay developed by members of or laboratory was performed to identify any CIV-positive clinical samples, as previously described [8]. Briefly, a 5-μl aliquot of RNA template was mixed with 20 μl of mixture containing iScript One-Step RT-PCR Kit reagents (BioRad; Hercules, CA), 80 nmol of probe, and 200 nmol of forward (5′ GAA CAC CGA TCT TGA GGC ACT C 3′) and 200 nmol of reverse (5′ GGC ATT TTG GAC AAA GCG TCT AC 3′) primers to amplify 144 bp of the conserved influenza A virus matrix gene. Water and viral transport medium were used as negative controls, while the positive control consisted of 10 TCID50 of A/canine/CO/224986/2006 (H3N8). An epMotion Ep5070p (Eppendorf; Hamburg, Germany) automated system was used to load 96-well plates before amplification and detection by Mastercycler Realplex (Eppendorf), using previously described conditions [8].

### Virus isolation

All real-time RT-PCR CIV-positive nasal swabs were stored at −80 °C until virus isolation could be performed. Aliquots of 150–200 μl thawed viral transport medium were injected under the illumination of an electronic egg candle into the allantoic cavities of three 10-day old embryonated hen eggs. In addition, 50 μl of nasal swab sample was inoculated into MDCK cells on 6-well plates in duplicate. Inoculation medium contained both antibiotic (gentamicin for the egg inoculation and penicillin and streptomycin for the cell culture inoculations) and antifungal (amphotericin B) agents, as previously described [8]. Each isolate was blind-passaged twice (for a total of three passages) using 200 μl of the previous passage and evaluated for CIV by hemagglutination inhibition (HI) assays. All first-passaged and HI-positive isolates were additionally tested for the presence of the influenza matrix gene by real-time RT-PCR.

### Sequencing

The full-length HA protein-coding region for each isolated virus was amplified by a two-step RT-PCR. Briefly, extracted viral RNA was reverse transcribed using SZAHA primers [29] and SuperScript™ III reverse transcriptase (Gibco/Invitrogen, Carlsbad, CA) before HA cDNAs were amplified by PCR using Platinum^®^ Taq DNA polymerase high fidelity (Gibco/Invitrogen). PCR products were obtained by direct cycle sequencing using ABI Big Dye (PE Applied Biosystems, Foster City, CA) and sequenced at the Proteomics and Metabolomics Facility at CSU (http://www.dnatools.com). Nucleotide sequences of RNA from the shelter dog study, as well as several Colorado and Wyoming CIVs isolated in our laboratory since 2006, were compiled de novo using Geneious Pro software [30] and deposited into GenBank under accession numbers JX235372 through JX235394.

### Evolutionary and antigenic analyses

Both nucleotide and amino acid alignments were assembled using the Kyoto University Bioinformatics Center’s multiple sequence alignment online tool (http://www.genome.jp/tools/clustalw/). All available H3N8 CIV full-length HA sequences published up until 25-Jun-2012 were downloaded from the Influenza Virus Resource, National Center for Biotechnology Information (NCBI) database. Additionally, HAs of viruses previously sequenced by members of our laboratory (GenBank accession numbers HQ917678–HQ917681) were included. In total, there were 62 sequences (19 new shelter, 9 Colorado and Wyoming strains previously isolated by members of our laboratory, and 34 published sequences). Potential antigenic regions were identified according to sites reported for human and equine influenza H3 viruses [17–21]. The phylogenetic tree was inferred by amino acid analysis based on maximum parsimony with 1,000 bootstrap repeats and rooted to EIV Pre-divergence strains using MEGA5 [31]. To determine selection pressure, the Datamonkey web server [32, 33] using the HyPhy software package [34] was utilized to evaluate the nucleotide sequences from 58 (17 shelter, 8 previously sequenced in our laboratory, and 33 published) isolates; four nucleotide sequences were identical to other isolates and were excluded from analysis. Because more than two mutations might segregate at a single site in viral populations [35], the mixed-effects model for evolution (MEME) [36] method was used, using the HKY85 nucleotide substitution bias model. This method accounts for episodic selection at each individual amino acid site, rather than at only the node or branch alone [36]. Additionally, to account for any false positives in the MEME method, the single likelihood ancestor counting method [37] was also used to confirm any sites found to be undergoing selection pressure by the MEME analysis.

## Results

### Virus isolation

Over 5,100 nasal swab samples were collected from shelter dogs entering and exiting the six participating humane shelters over the study period. Of these, 111 were positive upon real-time RT-PCR for the influenza matrix gene (Table 1). After three passages in both embryonated hen eggs and MDCK cells, 19 viruses (17 %) were isolated and their HA genes were sequenced. The majority of these (11/19) were grown in allantoic fluid only, while one was grown in cell culture only, and seven viruses grew in both culture systems. Most of the isolates were from the Colorado and New York shelters, although one virus each was isolated from the Florida and South Carolina shelters. Interestingly, all of the Colorado isolates were obtained in the first year of the study (2009–2010), while all of the New York viruses were from nasal swabs collected during study year two (2010–2011) (Table 2). Additionally, the South Carolina virus was collected during the CIV-outbreak that occurred in a South Carolina shelter during the first month of the study (December 2009). The other viruses listed in Table 2 were isolated from either pet dogs seen at the Colorado State University Veterinary Teaching Hospital or were collected from dogs residing at Colorado and Wyoming humane shelters during reported outbreaks of canine infectious respiratory disease from 2006 to 2007.

**Table 1:** Viruses isolated from dogs residing in humane shelters during December 2009 through January 2012

Shelter location	Samples collected	Swabs inoculated	Isolates sequenced
California	854	1	0
Colorado	1037	38	7
Florida	953	12	0
New York	863	37	11
South Carolina	708	23	1
Texas	771	0	0
TOTAL	5186	111	19

**Table 2:** Canine influenza viruses isolated from nasal swabs collected from dogs during 2006–2011

Strain	Date collected	Passage^a^	Location
A/canine/CO/224986/2006	06-Jun-06	C3	CSU VTH^b^
A/canine/CO/148902/2006	30-Oct-06	C2	Humane shelter
A/canine/CO/3/2006	28-Apr-06	C3	CSU VTH
A/canine/CO/224766/2006	24-May-06	C2	CSU VTH
A/canine/WY/86033/2007	14-Feb-07	E3; C3	Humane shelter
A/canine/CO/231256/2007	08-Mar-07	C6	Humane shelter
A/canine/WY/86955/2007	27-Feb-07	E1; C3	Humane shelter
A/canine/CO/2025974/2007	14-Mar-07	E1; C3	Humane shelter
A/canine/CO/234550/2009	27-Feb-09	C6	CSU VTH
A/canine/SC/89215/2009	05-Dec-09	E3	Humane shelter
A/canine/CO/850078/2009	31-Dec-09	E3	Humane shelter
A/canine/CO/861142/2010	03-Mar-10	E3	Humane shelter
A/canine/CO/861997/2010	09-Mar-10	E3	Humane shelter
A/canine/CO/863160/2010	20-Mar-10	E3	Humane shelter
A/canine/CO/866907/2010	10-Apr-10	E3	Humane shelter
A/canine/CO/884753/2010	21-Jul-10	C1	Humane shelter
A/canine/CO/886050/2010	27-Jul-10	C3	Humane shelter
A/canine/NY/6977983/2010	05-Sep-10	E3	Humane shelter
A/canine/NY/12370090/2011	12-Feb-11	E4	Humane shelter
A/canine/NY/13258337/2011	05-Jun-11	E3	Humane shelter
A/canine/NY/13454104/2011	30-Jun-11	E1	Humane shelter
A/canine/NY/13453967/2011	30-Jun-11	C1	Humane shelter
A/canine/NY/3821631/2011	06-Jul-11	E3	Humane shelter
A/canine/NY/13693247/2011	03-Aug-11	E1	Humane shelter
A/canine/NY/13752393/2011	12-Aug-11	E3	Humane shelter
A/canine/NY/13889381/2011	25-Aug-11	E3	Humane shelter
A/canine/NY/13949315/2011	01-Sep-11	E1	Humane shelter
A/canine/NY/13949306/2011	01-Sep-11	E2	Humane shelter

^a^Passage used for sequencing, either from cell culture (C) or embryonated hen eggs (E). Number following C or E indicates number of passages. When isolates were sequenced from both embryonated hen eggs and cell culture, the sequences were found to be identical

^b^Colorado State University Veterinary Teaching Hospital

### Genetic evolution of CIV

**Fig. 1:**
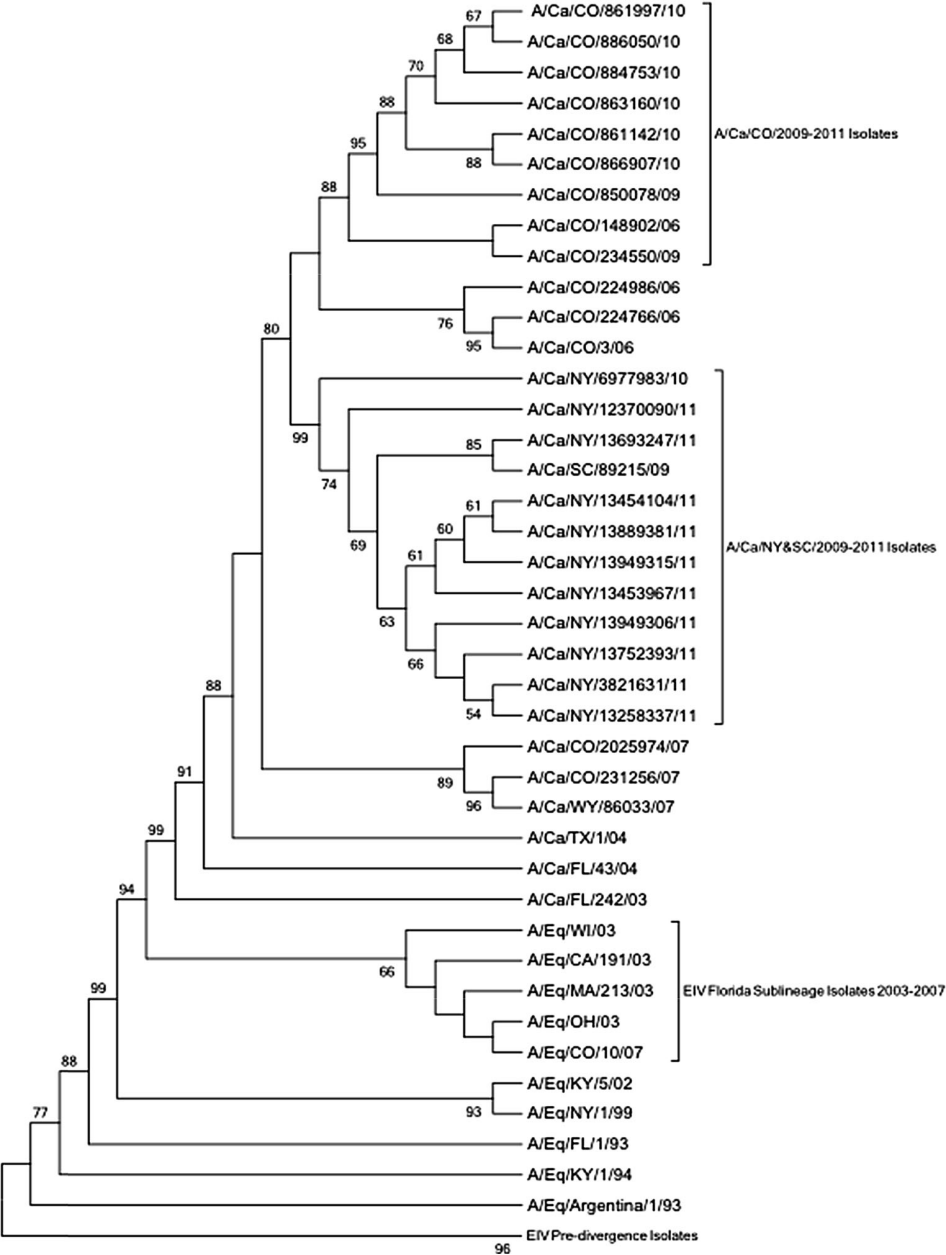
Phylogenetic comparison of canine and equine influenza virus H3 genes. Amino acid analysis was based on maximum parsimony with bootstrap analysis (values with >50 % consensus shown) and rooted to the EIV Pre-divergence strains

Genetic analysis of HA amino acid sequences suggests that recent CIVs appear to have evolved from earlier CIVs, as they have consistently maintained the originally described HA mutations from A/Ca/Florida/03 and A/Ca/Florida/04 [2, 3]. Additionally, phylogenetic analysis further suggests that CIV may be diverging regionally (Fig. 1), with the New York/South Carolina and the Colorado CIV isolates clustering separately from one another. Although viruses from these two regions share eight of the original nine adaptations from EIV and emerging CIV identified by Payungporn et al. [3], as well as the three HA substitutions reported by Rivallier et al. [14], there were an additional 12 amino acid differences among Colorado and New York CIV isolates (I58V, H75Q, D/E77D, I112V, G/E124G, K172K/E, Y174F, V223I, T/I242I, M268I, G/D464N/D, and L496I). Interestingly, six of these twelve newly identified differences were within H3 antigenic regions (Table 3). Moreover, nine amino acid differences appear among the viruses isolated during early infections in Colorado and Wyoming (2006–2007) and the isolates collected during the shelter study (2009–2010): D/E77E, S/L107L, V112I, G/E124G, F174Y, G/R218G, T/I242I, G/D464D, and E479K. This last site is of particular interest as it was one of the CIV substitutions identified when CIV was first detected in 2004 [2, 3].

**Table 3:** Amino acid differences within H3 antigenic regions among emerging CIV and contemporary H3N8 CIV isolates

H3 amino acid residue (antigenic regions A–E)	CIV FL 2004–2005 [14]	CIV CO and WY 2005–2007	CIV CO 2009–2010	CIV NY and SC 2009–2012
75^a^ (E)	H	H	H	*Q*
124 (A)	G	G/*E*	G	G
172 (D)	K	K	K	K/*E*
174 (D)	F	F	*Y*	F
216^a^ (D)	N	N	N	N/*H*
223 (D)	V	V	V	*I*
242 (D)	I	T/*I*	I	I
262^a^ (E)	T	T	T	*P*

Italicized entries denote consistent differences between FL (2004–2005), CO/WY (2005–2007), CO (2009–2010), and NY/SC (2009–2012) isolates

^a^Sites previously identified [2, 3, 14, 27]

The MEME analysis revealed five sites exhibiting evidence of episodic diversifying selection at a significance level of *p* < 0.1. These included H3 amino acid residues 107, 169, 216, 453, and 464. Site 107 has been reported to be involved in cellular immunity [38], while sites 169 and 216 are located in or near potential antigenic sites. Amino acid residues 453 and 464 are located on the highly conserved HA2 subunit of the HA protein. However, the single likelihood ancestor counting method only shared amino acid residue 464 as undergoing positive selection pressure, while residues 184, 449, 458, and 506 were considered undergoing negative selection pressure.

## Discussion

Since the emergence of CIV nearly 10 years ago, the virus has circulated in a number of dog populations, most notably in dogs residing in humane shelters [2, 7]. Indeed, CIV is routinely isolated from such facilities [8, 14, 27]. Whether this is because there are actually more CIV infections in shelter dogs compared to non-shelter dogs or there is more surveillance in this particular population is a matter under current study [28]. Nevertheless, the frequent isolation of CIVs in shelter dogs presents a unique opportunity to characterize genetic evolution and antigenic variation of a relatively novel influenza A virus “in situ.”

Results from these studies and others [14, 27] suggest that CIV might be diverging into two regionally distinct lineages: Colorado and New York. Indeed, the recent New York viruses and the one South Carolina isolate appear to cluster separately from CIVs isolated in Colorado and Wyoming (Fig. 1). Additionally, a number of CIV lineages have not been isolated recently. For example, isolates from a Wyoming animal shelter (A/canine/WY/86955/2007 and A/canine/WY/86033/2007) are not clustered with any of the newer Colorado isolates (Fig. 1), while, instead, recent Colorado CIVs are clustered with viruses isolated from dogs residing at other animal shelters located within Colorado. Despite the possible geographical divergence of CIV, the separate lineages might be due rather to temporal mutations, as the Colorado CIVs were isolated in 2009 and 2010, before the New York CIVs were isolated in 2010 and 2011. However, giving more weight to the regional divergence claim is the fact that the South Carolina CIV, which most resembles the New York CIVs, was isolated in 2009, before the Colorado CIVs were isolated.

The MEME analysis found five sites in the HA coding region undergoing episodic diversifying selection: 107, 169, 216, 453, and 464. Three of these sites (AA residues 107, 169, and 216) are located on the HA1 protein (HA sites 1 through 328), including one site located in an potential antigenic region, while two of these (residues 453 and 464) are on the HA2 protein (residues 330 through 550). The single likelihood ancestor counting method also identified 464 as undergoing positive selection pressure, suggesting that there are indeed amino acid changes occurring within the highly conserved HA2 protein. Although residue 107 was not identified by single likelihood ancestor counting analysis as undergoing selection pressure, substitutions at site 107 were reported in 2008 as a replacement of serine in the conserved EIV and early Florida CIV isolates by a proline in the A/canine/Jacksonville/2005 strain [3]. Site 107 appears to be part of a peptide region (AA residues 105-140) that might stimulate proliferation of T helper cells [38]. Isolates substituting serine at position 107 change the amino acid from one with a polar uncharged side chain to either one with a secondary amine (proline) or one with a hydrophobic side chain (leucine). However, recent isolates from both New York and Colorado show a leucine substitution at this site instead.

With eight amino acid substitutions within three potential antigenic regions, antigenic drift might also be occurring for the newer CIV isolates. However, it is important to note that E124 (site A) was only observed in two of the viruses isolated after the Wyoming 2006 outbreak (A/canine/CO/231256/2007 and A/canine/WY/86033/2007). Other mutations seen in only a few isolates included two clinical samples from the Colorado State University Veterinary Teaching Hospital with I242T (A/canine/CO/3/2006 and A/canine/CO/224766/2006) and two shelter CIVs with K172E (A/canine/SC/89215/2009 and A/canine/NY/13693247/2011). Despite these transient mutations, it appears that five consistent substitutions have occurred in the Colorado and New York isolates. In antigenic site D, two amino acid substitutions (N216H and V223I) were identified in the New York isolates. Also within site D, another substitution (F174Y) was seen in the CIVs isolated from the Colorado shelter during 2009–2010, while within antigenic site E, two amino acid residue mutations were seen in New York isolates (H75Q and P262T).

These consistent mutations among CIV isolates, despite passage number and culture system, suggest that CIV is indeed evolving and not an artificial result of in vitro selection pressure. Additionally, as the analyses presented here are based on genomic and antigenic sites described for human and equine H3 influenza A viruses [17–21], it is possible that the CIV HA has different antigenic regions that have yet to be defined. More research on CIV is warranted to confirm the structure of the CIV HA protein, map antigenic regions associated with virus neutralization and antigenic drift, and further investigate antigenic variation leading to CIV immune escape. Finally, one limitation of our study was the possible degradation of nasal swab samples between collection, transport, and processing, as well as the fact that sequencing directly from nasal swabs has, thus, far been unsuccessful. As samples were stored on-site at humane shelters before being shipped to the Colorado State University laboratory, infectious virus might have been compromised, which could account for our low number of isolates. Future studies utilizing virus isolation might want to find a quicker, yet practical, protocol for sending samples to the laboratory soon after collection.
